# Very Low-Volume, High-Intensity Interval Training Mitigates Negative Health Impacts of COVID-19 Pandemic-Induced Physical Inactivity

**DOI:** 10.3390/ijerph191912308

**Published:** 2022-09-28

**Authors:** Dejan Reljic, Annalena Eichhorn, Hans J. Herrmann, Markus F. Neurath, Yurdagül Zopf

**Affiliations:** 1 Hector-Center for Nutrition, Exercise and Sports, Department of Medicine 1, University Hospital Erlangen, Friedrich-Alexander University Erlangen-Nürnberg, 91054 Erlangen, Germany; 2 German Center Immunotherapy (DZI), University Hospital Erlangen, Friedrich-Alexander University Erlangen-Nürnberg, 91054 Erlangen, Germany; 3 Department of Medicine 1, University Hospital Erlangen, Friedrich-Alexander University Erlangen-Nürnberg, 91054 Erlangen, Germany

**Keywords:** exercise, health promotion, cardiometabolic health, lockdown, occupational health management, physical activity

## Abstract

Initially, we aimed to investigate the impact of a one-year worksite low-volume, high-intensity interval training (LOW-HIIT) on cardiometabolic health in 114 sedentary office workers. Due to the COVID-19 pandemic outbreak, LOW-HIIT was discontinued after 6 months and participants were followed up for 6 months to analyze physical activity/exercise behavior and outcome changes during lockdown. Health examinations, including cardiopulmonary exercise testing and the assessment of cardiometabolic markers were performed baseline (T-1), after 6 months (T-2, termination of worksite LOW-HIIT) and 12 months (T-3, follow-up). Cycle ergometer LOW-HIIT (5 × 1 min at 85–95% HR_max_) was performed 2×/week. For follow-up analyses, participants were classified into three groups: HIIT-group (continued home-based LOW-HIIT), EX-group (continued other home-based exercises), and NO-EX-group (discontinued LOW-HIIT/exercise). At T-2, VO_2max_ (+1.5 mL/kg/min, *p* = 0.002), mean arterial blood pressure (MAB, −4 mmHg, *p* < 0.001), HbA_1c_ (−0.2%, *p* = 0.005) and self-reported quality of life (QoL, +5 points, *p* < 0.001) were improved. At T-3, HIIT-group maintained VO_2max_ and QoL and further improved MAB. EX-group maintained MAB and QoL but experienced a VO_2max_ decrease. In NON-EX, VO_2max_, MAB and QoL deteriorated. We conclude that LOW-HIIT can be considered a promising option to improve cardiometabolic health in real-life conditions and to mitigate physical inactivity-related negative health impacts during lockdowns.

## 1. Introduction

The multiple benefits of regular physical activity and exercise for the prevention of numerous health problems and chronic diseases, including type 2 diabetes mellitus, cardiovascular disease (CVD) and several types of cancer, have been documented in a large number of studies [1,2,3,4]. Research has demonstrated, for example, that the degree of cardiorespiratory fitness (typically quantified as maximal oxygen uptake, VO_2max_), is a leading predictor of chronic disease and premature mortality—independent of other pre-existing risk factors, such as obesity, hypertension or smoking [5]. However, despite the overwhelming evidence of the health-protective effects of being regularly physically active, large proportions of the population in industrialized countries fail to achieve the recommended minimal amounts of physical activity in today’s world of increasing workload and technization [6]. The individual reasons for insufficient physical activity and non-participation in specific exercise routines are manifold and complex, but large-scale surveys conducted over the last decade have repeatedly shown that “lack of time” and “lack of social support” are among the major barriers for leading a physically active lifestyle and engaging in exercise-training programs [7,8,9]. Consequently, in recent years, the evaluation of the beneficial health effects of more time-efficient exercise types has increasingly become the subject of extensive research [10].

In this context, low-volume high-intensity interval training (LOW-HIIT), a particular time-efficient variation of the well-known interval training method, has emerged as a popular and effective exercise modality that has shown great potential in improving a broad range of health outcomes with substantially less time investment compared to other exercise methods, such as moderate-intensity continuous endurance training [11,12,13,14,15]. Recent studies from our group revealed, for example, that LOW-HIIT can effectively improve VO_2max_, cardiometabolic health outcomes and quality of life (QoL) in various populations, including sedentary healthy individuals [16], obese metabolic-syndrome patients [17,18,19,20] and advanced cancer patients [21]. Interestingly, data from our laboratory also indicate that participating in LOW-HIIT may improve physical activity behavior over the longer term as many individuals involved in our previous studies reported that they intended to continue engaging in interval training and/or other exercise modalities after termination of the LOW-HIIT intervention [16,18,19,21]. In line with our findings, it has been shown that previously physically inactive, healthy individuals who performed 10 weeks of LOW-HIIT in a gym setting sustained levels of physical activity during a 3-month follow-up period [22].

Thanks to the growing awareness among employers that physical inactivity is a major contributor to absenteeism (i.e., absence from the workplace due to illness or disability) and presenteeism (i.e., diminished productivity at work) and increasing evidence suggesting that promotion of physical activity at the workplace can contribute to improving the health status and work performance of workers [23,24,25,26], an expanding number of companies have begun to offer workplace exercise programs for their employees. Given that many people spend a large amount of their day time at work, the workplace may indeed represent an ideal setting to promote physical activity. In this context, LOW-HIIT, due to its high time efficiency, may be an interesting option for worksite exercise programs. However, the long-term effects of HIIT in real-world conditions outside of the laboratory (e.g., at the workplace) have only been scarcely investigated to date [27,28].

Thus, it was the initial aim of the present study to evaluate the impact of a one-year supervised LOW-HIIT program in a worksite setting on cardiometabolic health and self-reported outcomes in a cohort of previously sedentary office workers. The original project was intended as a longer-term and larger sample size follow-up study to a previously published pilot study by Reljic et al. [16], in which preliminary results indicated that as little as 28 min of LOW-HIIT per week effectively improved various aspects of cardiometabolic health and self-reported work ability in sedentary individuals within only 8 weeks. Based on our previous research [16,17,18,19,20], we initially hypothesized that LOW-HIIT performed under real-life conditions at the workplace over a period of one year would be an effective modality to induce beneficial long-term effects on cardiometabolic health including improvements in VO_2max_ and blood pressure and positive changes in self-reported outcomes, such as quality of life (QoL) and work ability.

However, due to the unexpected COVID-19 outbreak during our ongoing worksite intervention and the resulting sudden shift to home-office for all study participants, the study design was adjusted as follows: The supervised worksite program was discontinued after 6 months and participants received a post-intervention examination to evaluate the effects of the supervised worksite LOW-HIIT program (Study Part 1). Subsequently, participants were followed up for 6 months during lockdown and invited back for a third examination (Study Part 2). The aim of Study Part 2 was to analyze participants’ physical activity and exercise behavior during the pandemic situation and to re-examine changes in cardiometabolic and self-reported outcomes. Due to the novelty of the COVID-19 pandemic and a lack of previous data, Study Part 2 was rather exploratory and we did not define a precise hypothesis but we assumed that participants’ physical activity and exercise levels would significantly decline during lockdown, which would consequently have a negative impact on their cardiometabolic health and self-reported outcomes.

## 2. Materials and Methods

### 2.1. Experimental Design

This study was a 6 month pre-/post-intervention assessment with a subsequent 6-month follow-up period. Data from the whole cohort were compared in a pre- (baseline examination, T-1) versus post-intervention (after the premature termination of the supervised worksite LOW-HIIT program after 6 months, T-2) design. For follow-up analysis (T-3), participants’ self-reported, unsupervised exercise behavior during the lockdown period was evaluated and subsequently, three subgroups were classified as follows: (1) HIIT-group = participants who continued with LOW-HIIT at home; (2) EX-group = participants who discontinued LOW-HIIT but continued with other exercise modalities at home; and (3) NON-EX-group = participants who discontinued LOW-HIIT and did not participate in any other type of exercise. The primary outcome of this study was maximal oxygen uptake (VO_2max_). Secondary outcomes were further cardiometabolic health indices, anthropometric variables and self-reported outcomes as specified in detail below. All participants were fully informed about the objectives and procedures of the study, which was in accordance with the Helsinki Declaration, and provided written consent before enrolment. The study protocol was approved by the Ethics Committee of the Medical Faculty of the Friedrich-Alexander University Erlangen-Nürnberg (approval number: 123_19B).

### 2.2. Participants

A total of 114 (44% females) sedentary office workers employed at a large company close to our Research Center volunteered to participate in this study. Participants were recruited via flyers and intranet postings of the cooperating company. All interested workers were able to contact our research staff by mail or phone to discuss the criteria for study participation. If the criteria were formally met, the interested candidates were given an appointment for the baseline examination. The inclusion criteria were as follows: female and male office workers aged ≥18 years, a self-reported predominantly sedentary lifestyle as previously defined by the American College of Sports Medicine [29] (i.e., no specific exercise training and engaging in less than 30 min of moderate physical activity three times per week) over at least 1 year prior to the study and no plans to start another specific exercise program during the intervention period. The exclusion criteria were as follows: clinical diagnosis of heart disease, cancer, severe orthopedic conditions, injuries and other conditions that might prohibit safe participation in an exercise program, and pregnancy. As no specific guidelines on exercise after a COVID-19 infection were available at the time, all participants with a previous positive COVID-19 diagnosis were also excluded from the study to ensure participant safety and to rule out unknown bias on outcomes. Participants were asked to maintain their usual lifestyle patterns throughout the intervention period to minimize any potential confounding effects of changes in diet and daily physical activity.

### 2.3. Health Examinations

The baseline health examination (T-1) was conducted 1–2 weeks before the onset of the worksite LOW-HIIT intervention at our Research Center. The second (post-intervention, T-2) and third (follow-up, T-3) examination was carried out at an equal day time to minimize potential circadian effects. For each examination, participants were asked to arrive in an overnight-fasted state and to refrain from alcohol and vigorous physical activity in the preceding 24 h. All measurements were strictly standardized and carried out in the order as described in the following.

#### 2.3.1. Blood Pressure Measurements

After arrival to the laboratory, participants were first asked to empty their bladder if necessary and, subsequently, to rest in a seated position for 5 min. Thereupon, resting blood pressure was measured two times on each arm using an automatic upper-arm blood pressure analyzer (M5 professional, Omron, Mannheim, Germany). As recommended by recent guidelines [30], the values of the arm with the higher blood pressure were averaged and recorded for further analysis. Moreover, mean arterial blood pressure (MAB) was calculated as follows:

MAB = diastolic blood pressure + (1/3[systolic blood pressure − diastolic blood pressure])

#### 2.3.2. Blood Sampling

Blood samples were collected through venipuncture of the antecubital arm vein using a disposable cannula (S-Monovette, Sarstedt, Nürmbrecht, Germany). Blood analyses were conducted at the laboratories of the University Hospital Erlangen and included fasting glucose, triglycerides, total cholesterol, low-density lipoprotein cholesterol (LDL-C), high-density lipoprotein cholesterol (HDL-C), which were measured photometrically (Clinical Chemistry Analyzer AU700 or AU5800, Beckman Coulter, Brea, CA, USA), and glycated hemoglobin A1c (HbA_1c_) using turbidimetric immuneassays (COBAS Integra 400, Roche Diagnostics, Mannheim, Germany, respectively).

#### 2.3.3. Body Composition Measurement

Body composition measurements were performed with a multifrequency segmental bioelectrical impedance analysis device (seca mBCA 515, Seca, Hamburg, Germany), which has been previously validated against magnetic resonance imaging [31]. Waist circumference was measured in upright position to the nearest millimeter, at the approximate midpoint between the lower margin of the last palpable rib and the upper iliac crest along the midaxillary line, using a measuring tape.

#### 2.3.4. Cardiopulmonary Exercise Testing (CPET)

Participants performed a standardized exercise test on a stationary electronically braked cycle ergometer (Corival cpet, Lode, Groningen, Netherlands) to determine VO_2max_, maximal power output (W_max_) and maximal heart rate (HR_max_). After a 1 min period of familiarization, the ergometer load was set at 50 W, then continuously increased in a ramp manner by 1 W every 5 s (25 W/2 min) in female participants and by 1 W every 4 s (30 W/2 min) in male participants, respectively, until volitional exhaustion. Using this protocol, exhaustion was typically reached within 8–12 min as previously recommended for CPET examinations [32]. A 12-lead ECG system (custo cardio 110, custo med, Ottobrunn, Germany) was used to record HR at rest and continuously during the exercise test. An open-circuit breath-by-breath spiroergometric system (Metalyzer 3B-R3, Cortex Biophysik, Leipzig, Germany) was used to measure oxygen uptake (VO_2_) and carbon dioxide output (VCO_2_) during rest and exercise. The criteria to assume that maximal effort (i.e., VO_2max_) was reached were at least two of the following [33]: a VO_2_ plateau, ≥ 90% of age predicted HR_max_ (APHR_max_, using the equation: 220–age), maximal respiratory exchange ratio (RER_max_)  ≥  1.10, and maximal rate of perceived exertion (RPE_max_)  ≥  19 at exhaustion using the 6–20 Borg scale [34]. Additionally, power output at the ventilatory threshold (W_VT_) was determined according to the V-Slope method (VCO_2_/VO_2_) [35].

#### 2.3.5. Self-Reported Outcomes

For the assessment of self-reported outcomes, participants completed standardized questionnaires, which were all previously validated in the German language. Health-related quality of life (QoL) was assessed using the EuroQol Group questionnaire (EQ-5D-5L), which consists of two measures: a visual analogue scale (EQ-VAS, 0–100 points, with higher values reflecting better QoL) and a descriptive system of 5 health-related QoL-dimensions (mobility, self-care, usual activities, pain/discomfort, anxiety/depression, each classified in 5 severity levels), which are converted to a single index value (EQ-5D-5L) [36]. Subjective work ability was examined using the Work Ability Index (WAI), which is composed of several dimensions, including work environment, skills and individual health. The total score ranges from 7 to 49, with higher values reflecting better subjective work ability [37]. The Perceived Stress Questionnaire (PSQ) was used to analyze stress perception, including the 4 components “worries”, “tension”, “joy”, and “demands”, with higher values reflecting higher stress levels (except for the sub-dimension “joy”) [38]. At T-2, participants additionally provided a personal evaluation sheet, including their intentions to further engage either in exercise in general or specifically in LOW-HIIT, respectively.

#### 2.3.6. Monitoring of Daily Physical Activity

One week prior to each examination, participants were asked to log their daily physical activity (including, e.g., housework, cycling to work) using an activity logbook. The logbook consisted of an instruction page and log pages for each recording day over a period of 1 week. Participants were instructed to make daily notes regarding their sedentary times and all physical activities, including type, time and duration of the activities. Similar logbooks have been previously used in several other studies [39,40]. Data from the logbooks were used to calculate the respective metabolic equivalent (MET) intensity levels of each reported daily-life physical activity, which were classified as light (<3 METs), moderate (3–6 METs), or vigorous (>6 METs), as suggested by Ainsworth et al. [41], and subsequently, the average MET hours per week were estimated. During the follow-up period, participants were additionally instructed to record all specific home-based exercise activities (including LOW-HIIT) with the help of a training diary.

#### 2.3.7. Monitoring of Nutrition

During the week prior to each examination, participants were advised to record their daily nutritional intakes over three consecutive days using standardized food records (Freiburger Ernährungsprotokoll; Nutri-Science, Freiburg, Germany). Average calorie and macronutrient intakes were analyzed using a specific nutrition analysis software (PRODI 6 expert, Nutri-Science, Freiburg, Germany).

#### 2.3.8. LOW-HIIT Program

The worksite LOW-HIIT program took place in a training room within facilities of the cooperating company and performed on mechanically-braked, indoor spinning bikes (A.C Performance Plus Bike, Schwinn Bicycle Company, Vancouver, WA, USA) as group classes. All exercise sessions were supervised by certified sports- or physiotherapists, who were trained in implementing the specific LOW-HIIT protocol. The LOW-HIIT protocol was similar to the protocol developed and previously described in detail by Reljic et al. [16]. Briefly, the protocol consisted of a short 2 min warm-up phase, 5 interval bouts of 1 min duration at an intensity corresponding to 85–95% of maximum heart rate (HR_max_) interspersed with 1 min recovery periods of low intensity pedaling and a concluding 3 min cool-down phase (total time effort per session: 14 min). The minimum target exercise intensity (heart rate) to be reached during the single intervals was 85–90% HR_max_ during week 1–4 week, and 90–95% HR_max_ from week 5, and further on. To reach their individually prescribed target heart rate for each interval bout, participants were instructed to increase the pedal cadence and/or the load resistance. To monitor heart rate in real-time during each session, participants were provided with a chest strap heart rate monitor (Polar H7 heart rate sensor, Polar Electro Oy, Kempele, Finland). Heart rate values were recorded continuously throughout each exercise session and, subsequently, analyzed using a specific heart rate monitoring system (Polar Team, Polar Electro Oy, Kempele, Finland). Participants were asked to attend two weekly LOW-HIIT sessions with a minimum of 1-day rest between sessions. To maximize attendance among participants, a total of 24 possible sessions were offered per week (Monday to Friday, distributed over several time slots during the day). The exercise sessions could be scheduled individually with the use of an electronic course planner, in which the participants could sign up for up to 10 min before the start of the course.

#### 2.3.9. Statistical Analysis

All statistical analyses were conducted using SPSS version 24.0 (SPSS Inc., Chicago, IL, USA). In the first step, data distribution was checked using the Shapiro–Wilk test. Pre- (T-1) and post-intervention (T-2) data of the whole sample were compared using paired t-tests. Additionally, subgroup analyses were performed to check whether gender and weight status (i.e., normal range, BMI: <25.0 kg/m^2^; pre-obese, BMI: BMI: 25.0–29.9 kg/m^2^; and obese, BMI: ≥30.0 kg/m^2^) had any influence on changes in the primary outcome VO_2max_, cardiometabolic markers and QoL. For follow-up analysis (T-3), participants were divided into 3 subgroups: (1) participants who continued with LOW-HIIT at home (HIIT-group), (2) participants who did not continue LOW-HIIT but engaged in any other type of exercise (EX-group), and (3) participants who neither continued LOW-HIIT nor engaged in any other type of exercise (NO-EX-group). Subsequently, a 2 × 3 repeated-measures ANOVA was conducted to test for main effects of time (T-2 vs. T-3) and group (HIIT vs. EX vs. NO-EX), and interaction between both factors, followed by Holm–Sidak post hoc tests where appropriate. The homogeneity of variance was checked with the Levene’s test. For non-normally distributed data, log-transformation was used and the same analyses were applied to the transformed values. If log-transformation did not lead to data normalization (i.e., serum glucose, triglycerides and HbA_1c_, all self-reported outcomes except for WAI), Wilcoxon tests and Friedman two-way analyses by ranks, respectively, were conducted. Effect sizes were calculated using Cohen’s d or r, partial eta-squared (ɳp^2^) or Kendall’s coefficient of concordance (W), where appropriate. For all analyses, the significance level was set at *p*  <  0.05. All data are reported as means  ±  standard deviation (SD). Significant changes between T-1 and T-2 and between T-2 and T-3 are presented with 95% confidence intervals (95% CI).

## 3. Results

### 3.1. Study Flow

**Figure 1:**
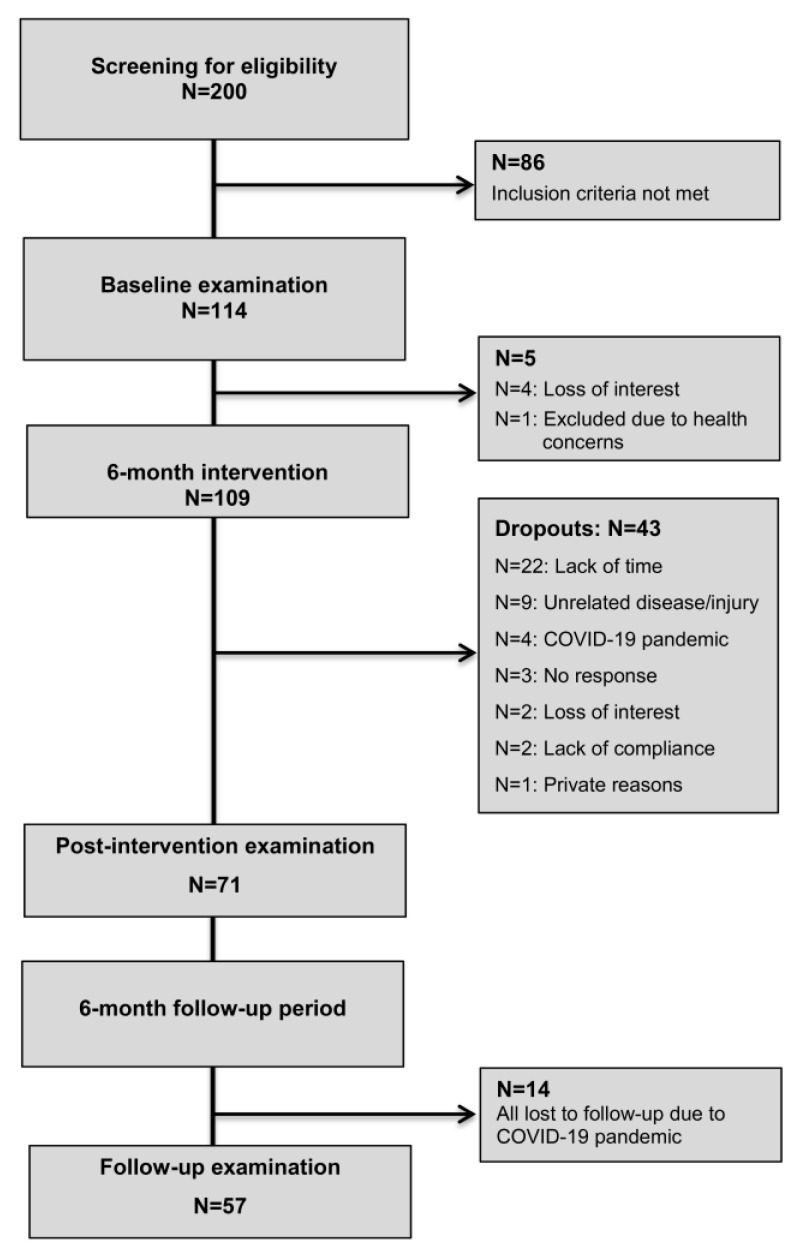
Study flow chart.

A total of 200 workers responded to the study call and expressed interest in participating in the LOW-HIIT program. A total of 114 participants (44% females, age: 48 ± 10 yrs) fulfilled the inclusion criteria and were invited to the baseline health examination after the initial phone screening. One participant was not able to participate due to health concerns detected during the health examination and three individuals lost interest in participation after the baseline examination. Thus, a total of 110 participants started the LOW-HIIT program. A total of 43 participants dropped out within the first 6 months. The specific reasons for dropout are shown in Figure 1. There were no significant differences in the main baseline values between participants who dropped out (age: 44 ± 10 yrs, BMI: 26.9 ± 5.3 kg/m^2^, VO_2max_: 33.0 ± 6.7 mL/min) and those who completed the intervention period (Table 1 and Table 2), except for the average age, which was lower in the dropout group (*p* = 0.008). After the premature termination of the supervised training intervention due to the COVID-19 pandemic, 72 participants (39% females, age: 50 ± 10 yrs) showed up for the post-intervention examination (T-2). Finally, a total of 57 participants (33% females) appeared for the follow-up examination, 12 months after the onset of the study (T-3). No significant effects of gender and weight status on changes in the primary outcome VO_2max_, cardiometabolic markers and QoL were found, and therefore, the results of all subgroups were considered together in all analyses.

### 3.2. Study Part 1: Post-Intervention Data

Over the intervention period of 6 months, participants completed on average 1.5 ± 0.2 LOW-HIIT sessions per week, which was lower than projected (i.e., 2 sessions/week). The average peak heart rate values reached during the interval bouts corresponded to 95 ± 3% of HR_max_, indicating that the prescribed exercise intensity was well achieved. The average heart rate during the complete exercise sessions (including warm-up, intervals, recovery phases and cool-down) corresponded to 82 ± 4% of HR_max_. No adverse events occurred during the whole intervention period. As intended, energy and macronutrient intakes remained stable throughout the intervention period. Daily calorie and macronutrient intakes averaged 2179 ± 738 kcal, 228 ± 95 g carbohydrates, 87 ± 35 g fat and 86 ± 34 g protein at T-1, and 2235 ± 888 kcal, 230 ± 91 g carbohydrates, 89 ± 46 g fat and 94 ± 42 g protein at T-2, respectively.

#### 3.2.1. Post-Intervention Cardiorespiratory Fitness

Participants experienced significant improvements in relative (+1.5 mL/kg/min, 95% CI 0.6 to 2.3 mL/kg/min, *p* = 0.002, d = 0.19) and absolute VO_2max_ (+0.17 L, 95% CI 0.05 to 0.19 L, *p* = 0.001, d = 0.13), relative (+0.3 W/kg, 95% CI 0.2 to 0.3 W/kg, *p* < 0.001, d = 0.38) and absolute W_max_ (+22 W, 95% CI 17 to 26 W, *p* < 0.001, d = 0.36) and W_VT_ (+41 W, 95% CI 31 to 51 W, *p* < 0.001, d = 0.95). Pre- and post-intervention data of all cardiorespiratory fitness variables are shown in Table 1.

#### 3.2.2. Post-Intervention Body Composition

There were no significant changes in any body composition variable. Pre-/post-intervention data are shown in Table 2.

#### 3.2.3. Post-Intervention Blood Pressure and Blood Chemistry

Resting heart rate (−18 b/min, 95% CI −24 to 14 b/min, *p* < 0.001, d = −1.20), diastolic blood pressure (−5 mmHg, 95% CI −7 to −3 mmHg, *p* < 0.001, d = −0.63) and MAB (−4 mmHg, 95% CI −6 to −2 mmHg, *p* < 0.001, d = −0.40) were significantly decreased at T-2. Moreover, there was a significant decrease in HbA_1c_ (−0.2%, 95% CI −0.1 to −0.4%, *p* = 0.005, r = −0.27). Pre-/post-intervention data of blood pressure data and all blood values are shown in Table 2.

#### 3.2.4. Post-Intervention Self-Reported Outcomes

Between T-1 and T-2, there were significant improvements in health-related QoL (EQ-VAS, +5 score points, 95% CI 2 to 7 score points, *p* < 0.001, r = 0.45). Pre-/post-intervention data of all self-reported outcomes are reported in Table 3. The major barriers to regular exercise reported by participants at T-1 were “lack of time” (67%), “lack of motivation” (56%) and “health problems” (15%). At T-2, 75% of participants reported that the LOW-HIIT program was helpful to overcome the previously perceived major barrier to exercise (lack of time) and 79% of participants stated that they intended to further engage in LOW-HIIT after termination of the study.

### 3.3. Study Part 2: Follow-Up Data

#### 3.3.1. Physical Activity Behavior and Diet during Lockdown

At T-3, 19 participants (HIIT-group, 21% females) reported that they have continued with LOW-HIIT using an own cycle ergometer at home. On average, LOW-HIIT was performed 1.6 ± 0.7 times per week. Twelve participants (EX-group, 33% females) reported that they have discontinued LOW-HIIT (N = 11: no cycle ergometer available at home, N = 1: lack of motivation) but performed other types of exercise at home, including moderate-intensity continuous jogging (N = 2), walking (N = 8) and yoga (N = 2). Other exercise modalities were performed 1.8 ± 0.6 times per week for an average duration of 23 ± 7 min per session (average time commitment per week: 42 ± 16 min). Twenty-six participants (NO-EX-group, 42% females) discontinued LOW-HIIT and did not perform any other type of exercise during lockdown. The reported reasons for discontinuation of LOW-HIIT and other types of exercise were: COVID-19 pandemic (N = 16), no ergometer available (N = 8) and lack of time (N = 2). The analysis of the activity logbooks revealed that habitual physical activity was generally very low in study participants during all recording periods. None of the participants met the physical activity guidelines of at least 150 min moderate-intensity activity per week [42]. Daily physical activities (excluding LOW-HIIT and other exercise types), especially moderate-intensity activities (e.g., cycling to work), and respective MET hours per week decreased significantly during lockdown in all groups. There were no significant differences in mean age and habitual physical activity between the three groups and no significant within- and between-group differences in calorie and macronutrient intakes, except for fat consumption, which was significantly higher in the HIIT- compared to the EX-group (*p* = 0.034). Group-specific physical activity and nutritional data during the weeks prior to T-2 and T-3 are displayed in Table 4.

#### 3.3.2. Follow-Up Cardiorespiratory Fitness

There were significant group-by-time interactions for relative (*p* < 0.001, ή^2^ = 0.34) and absolute VO_2max_ (*p* < 0.001, ή^2^ = 0.37). Significant main-time effects were found for relative (*p* < 0.001, ή^2^ = 0.30) and absolute VO_2max_ (*p* < 0.001, ή^2^ = 0.37), relative (*p* < 0.001, ή^2^ = 0.36) and absolute W_max_ (*p* < 0.001, ή^2^ = 0.42) and W_VT_ (*p* < 0.001, ή^2^ = 0.49). Post hoc tests revealed that relative and absolute VO_2max_ and W_VT_ only remained stable in the HIIT-group, while both the EX-group (relative VO_2max_: −3.7 mL/kg/min, 95% CI −5.7 to −1.7 mL/kg/min, *p* < 0.001, d = −0.54, absolute VO_2max_: −0.26 L, 95% CI −0.40 to −0.13 L, *p* < 0.001, d = −0.39, and W_VT_: −36 W, 95% CI −47 to −24 W, *p* < 0.001, d = −0.85) and the NON-EX group (relative VO_2max_: −5.8 mL/kg/min, 95% CI −7.6 to −4.1 mL/kg/min, *p* < 0.001, d = −0.72, absolute VO_2max_: −0.51 L, 95% CI −0.64 to −0.38 L, *p* < 0.001, d = −0.58, and W_VT_: −41 W, 95% CI −59 to −24 W, *p* < 0.001, d = −0.89) experienced significant decreases in cardiorespiratory fitness measures during the follow-up period (Table 5).

#### 3.3.3. Follow-Up Body Composition

In all three groups, there were no significant changes in body composition during the follow-up period. Group-specific data for T-2 and T-3 are shown in Table 6.

#### 3.3.4. Follow-Up Blood Pressure and Blood Chemistry

There were significant group-by-time interactions for systolic blood pressure (*p* = 0.002, ή^2^ = 0.15) and MAB (*p* = 0.013, ή^2^ = 0.18). Significant main-time effects were found for resting heart rate (*p* < 0.001, ή^2^ = 0.20), systolic (*p* < 0.001, ή^2^ = 0.31) and diastolic blood pressure (*p* < 0.001, ή^2^ = 0.37). In the HIIT-group, systolic blood pressure (−9 mmHg, 95% CI −15 to −2 mmHg, *p* = 0.008, d = −0.75), diastolic blood pressure (−4 mmHg, 95% CI −8 to 0 mmHg, *p* = 0.029, d = −0.63) and MAB (−4 mmHg, 95% CI −8 to 0 mmHg, *p* = 0.032, d = −0.47) further decreased during the follow-up period. MAB remained stable in the EX-group, while in the NO-EX-group, MAB (+3 mmHg, 95% CI +6 to +3 mmHg, *p* = 0.035, d = 0.40) and resting heart rate (+10 b/min, 95% CI +6 to +14 b/min, *p* < 0.001, d = 1.0) increased again significantly during lockdown. No significant changes occurred in any of the analyzed blood variables between T-2 and T-3 in all three groups. Group-specific data of all blood pressure and blood chemistry values are shown in Table 6.

#### 3.3.5. Follow-Up Self-Reported Outcomes

There was a significant main-time effect for the EQ-VAS score (*p* = 0.017, W= 0.10). Post hoc tests revealed that in the HIIT- and EX-group, EQ-VAS remained unchanged between T-2 and T-3, while the NO-EX-group experienced a significant decrease in the score during the follow-up period (−5 score points, 95% CI −9 to −1 score points, *p* < 0.001, r = 0.45). Pre-intervention and follow-up data of all self-reported outcomes are reported in Table 7.

## 4. Discussion

Originally designed as a one-year, workplace-based, exercise intervention for previously sedentary office workers, this study fell in the midst of the unexpected COVID-19 pandemic, requiring the project to be adjusted and divided into two sections: (1) a 6-month supervised worksite LOW-HIIT intervention period, and (2) a subsequent 6-month follow-up period during lockdown. The main findings of the study were as follows: (i) as little as 2 weekly sessions of worksite LOW-HIIT (<30 min time effort per week) significantly improved several cardiometabolic health indices, including VO_2max_, blood pressure and HbA_1c_ as well as self-reported QoL within 6 months, (ii) participants who discontinued LOW-HIIT and did not practice any other type of exercise during 6 months of COVID-19 related lockdown experienced a significant deterioration in VO_2max_, blood pressure and QoL, (iii) participants who discontinued LOW-HIIT but regularly performed other types of exercise maintained blood pressure values and QoL but experienced a significant decrease in VO_2max_, and (iv) participants who continued LOW-HIIT during lockdown maintained VO_2max_ and QoL and further improved blood pressure values.

### 4.1. Study Part 1

#### 4.1.1. Effects of the Worksite LOW-HIIT Intervention of Physiological Outcomes

As expected, the supervised, worksite-based, LOW-HIIT program improved various aspects of cardiometabolic health in our study participants. Previous studies from our group [16,17,18,19,20], applying the same LOW-HIIT protocol in sedentary but otherwise apparently healthy individuals and in obese metabolic syndrome patients, have also found significant improvements in cardiorespiratory fitness outcomes, blood pressure, glycemic control and self-reported outcomes including QoL. The results of the present study underpin our previous findings and reports from other research groups [10,11,15], indicating that brief, low-volume exercise modalities can be an effective and time-efficient approach to provide a broad range of health benefits in various populations. Referring to the call for more “real-world” HIIT studies over longer periods of time [27,28], the first part of our study expands the knowledge in this research field by showing that the health-related benefits associated with LOW-HIIT appear to be robust in longer-term authentic conditions outside of strictly standardized laboratory settings.

In this context, it is also important to note that no adverse events occurred during the whole intervention period and the majority of participants (79%) reported that they enjoyed the exercise program and intended to continue engaging in LOW-HIIT, indicating that this type of exercise is feasible and well accepted by previously sedentary individuals in real world conditions. The dropout rates (~24%, excluding those who dropped out due to the COVID-19 pandemic or other unrelated diseases/injuries) were lower compared to those frequently reported in the literature for other (more traditional) exercise interventions (30–50%) [43,44,45,46,47], but higher than in our pilot study (8%) [16] and other (LOW-)HIIT studies with previously untrained individuals (~18%) [48]. This finding could be due to the longer intervention duration (6 months) compared to previous (LOW-)HIIT studies (average duration: ~9 weeks) [48] and the fact that a larger proportion of our study participants who dropped out were involved in frequent business travels, which may have hampered longer-term regular participation in the training sessions.

The average increase in VO_2max_ in the present study (+1.5 mL/kg/min) was smaller compared to the values observed in our previous studies (ranging from 3.0 to 7.1 mL/kg/min) [16,17,18,19,20], which could be due to the low weekly exercise frequency/volume (i.e., ~1.5 sessions/week corresponding to 21 min of exercise/week). Compared to our previous studies (mainly conducted in laboratory settings), the present real-world intervention was less strictly standardized (including the mandatory compliance with 2 weekly exercise sessions). General physical activity guidelines typically recommend an exercise frequency of at least 3 sessions per week to provide adequate health benefits [49]. Although we [16,17,18,19,20,21] and others [50] have demonstrated that 2 sessions of targeted exercise per week may be sufficient to induce physiological adaptations that are linked to improved health, our present data suggest that less than 2 weekly sessions appear to be associated with smaller improvements in cardiorespiratory fitness compared to higher exercise frequencies. Moreover, it is well established that there is strong heterogeneity in VO_2max_ responses to training programs [51], which could also explain the differences to previous studies to some extent. However, given that each 1 mL/kg/min increase in VO_2max_ has been linked to a reduction in CVD-related mortality by 9% [52], the achieved VO_2max_ improvement in the present study can still be considered to be clinically meaningful. This is an important finding since lack of time is one of the most frequently reported barriers to engage in regular exercise, and the present findings suggest that VO_2max_ can effectively be improved with an average weekly time commitment of as little as 21 min. Additionally it is to note that we found a substantial increase in power output at the ventilatory threshold, which is an important submaximal indicator of cardiorespiratory fitness and recognized as a key marker of one’s ability to perform moderate-intensity activities of daily living for a longer duration [53].

Reductions in blood pressure following LOW-HIIT have been a consistent finding in the literature [54], including our own studies, which may be related to exercise-induced improvements in vascular function [55]. The observed mean reduction in diastolic blood pressure by 5 mmHg is very likely to provide a clinically significant benefit since each blood pressure decrease of 5 mmHg has been reported to reduce the risk of CVD and stroke by 22% and 41%, respectively, [56]. In this context, it is important to note that the average baseline blood pressure values of our participants were in the upper normal range (i.e., 131/90 mmHg), indicating a preventive anti-hypertensive effect of LOW-HIIT in normotensive individuals. The significant decline in HbA_1c_ levels by 0.2% is in line with our previous findings [20] and can also be regarded meaningful as changes in glucose control of this magnitude have been considered to lower premature mortality risk by 10% [57].

#### 4.1.2. Effects of the Worksite-Based, LOW-HIIT Intervention of Self-Reported Outcomes

Apart from physical health benefits associated with physical activity, there is a body of evidence demonstrating that exercise [58], including HIIT [59], can also improve a variety of subjective measures, such as wellbeing and QoL. In line with previous research from our group [18,19], we found a significant improvement in QoL following LOW-HIIT. Compared to the individuals included in our previous studies, who were obese metabolic syndrome patients with a markedly diminished self-reported baseline QoL, the participants of the present study exhibited a higher mean pre-intervention EQ-VAS score (78 points) in relation to the general population (71 points) [36]. Thus, our data suggest that even in individuals with high initial EQ-VAS scores, subjective QoL can be further improved in response to LOW-HIIT. Although this was not specifically investigated in our study, the improvement in QoL may be a result of physiological as well as psycho-social adaptations [60]. The physiological mechanisms could include improved cerebrovascular function [61] and neuronal effects, such as increased release of neurotransmitters and brain-derived neurotrophic factors [62]. At the psycho-social level, it is conceivable that engaging in LOW-HIIT improved participants’ physical self-perception [63]. By contrast, in comparison to our preceding studies [16,18], we did not find an increase in self-reported work ability, which could possibly be attributed to the restructuring of working conditions due the emerging COVID-19 pandemic (in particular the shift to home-office).

### 4.2. Study Part 2

Participants’ Exercise Behavior and Changes in Study Outcomes during Lockdown

The sudden outbreak of the COVID-19 pandemic has led to profound changes in many facets of society and culture, including lockdowns, social distancing, closure of cultural and sports facilities and the widespread implementation of home-office. One of the negative side effects of the pandemic was a substantial decline in physical activity in many countries worldwide [64,65,66]. A descriptive study has reported, for example, that globally, there was a ~27% decrease in mean daily steps within 30 days after declaration of the pandemic [67]. More specifically, a larger-scale representative survey involving more than 1000 German adults has shown, for example, that 31% of Germans significantly reduced their leisure time sport and exercise activities at the beginning of the COVID-19 pandemic [68]. Similarly, another longitudinal study assessing the data of almost 3000 people living in different Alpine regions (including Germany, Austria and Italy) revealed that 42.5% of all participants reduced their frequency of physical activity during the first lockdown. [69]. This recent trend is alarming because the reduction in physical activity and exercise due to constraints from the COVID-19 pandemic not only substantially contributes to the widespread inactivity-related health problems, but is also associated with the development of psychological problems such as stress, anxiety and depression [70,71] and is linked with an increased risk of developing a severe COVID-19 disease course [72]. Paradoxically, it has been suggested that regular exercise, including HIIT, may have a preventive role across the COVID-19 pandemic and reduce infection rates and diseases severity [73].

In line with these data, 46% of our study participants who showed up for the follow-up examination reported to have stopped any sports activities during 6 months of lockdown. The significant drop in VO_2max_ observed in the NON-EX group at T-3 by 5.8 mL/kg/min is in accordance with the literature, showing that prolonged periods of physical inactivity result in a gradual decline in cardiorespiratory fitness [74]. Given the reported associations between participation in endurance exercise [75], the degree of VO_2max_ [76] and blood pressure values, it is not a surprising finding that the NON-EX-group experienced a significant increase in MAB during the 6-month follow-up period. Moreover, although we did not detect any significant changes in self-reported perceived stress among participants, it cannot be ruled out that increased mental strains during the pandemic may have contributed to the elevation in participants’ blood pressure [77]. Accordingly, we found a significant reduction in self-reported QoL in the NON-EX-group, which is in line with the literature, reporting a significant decline in QoL during the COVID-19 pandemic in more than the half of participants of a population-based sample in Germany [78].

In contrast, participants who continued with LOW-HIIT or other exercise types, maintained EQ-VAS scores, which underpins the well-established association between a physically active lifestyle and self-reported QoL [79]. It is noteworthy, however, that only the HIIT-group maintained VO_2max_ during the follow-up period, while in the EX-group, we found an average decline of 3.7 mL/kg/min between T-2 and T-3. It is well established that more intense exercise is more effective at increasing/maintaining VO_2max_ [80] and thus, the observed difference between both groups is plausible as participants in the EX-group reported to have exclusively performed moderate-intensity aerobic training or other non-vigorous activity types. Moreover, this finding indicates that occasional moderate-intensity exercise at low frequency (i.e., ~1.8 sessions/week and ~42 min total volume/week, respectively) was not enough to compensate an otherwise physically inactive lifestyle when it comes to preserve VO_2max_. However, our data suggest that as little as 2 weekly sessions of home-based, unsupervised LOW-HIIT (<30 min time effort/week) performed over 6 months appear to be sufficient to counteract the negative impact of physical inactivity in daily life during lockdown on cardiorespiratory fitness and, concomitantly, to further improve blood pressure values in sedentary office workers. Therefore, home-based LOW-HIIT can be regarded as a feasible, time-efficient and effective exercise modality to maintain/improve cardiometabolic health and QoL during COVID-19 induced lockdown. Since it cannot be ruled out that renewed lockdowns or other restrictions could possibly occur again in the pandemic, which remains ongoing, LOW-HIIT may, therefore be particularly an interesting exercise option for individuals with time restrictions who wish to maintain their physical fitness at home.

### 4.3. Limitations

We point out some limitations of our study that should be taken into account. First, we only recruited previously sedentary but otherwise healthy individuals. Thus, the results of this investigation cannot be generalized to other populations, in particular individuals with health conditions or pre-existing risk factors. In this context, we highlight that a proper medical examination should be conducted prior to performing (particularly home-based) LOW-HIIT to ensure safe exercise, as generally recommended prior to starting a training program after longer periods of inactivity.

Second, we note that self-reported outcomes, such as QoL, may be biased by several factors, such as social desirability, conscientious responses or lack of memory. Additionally, questionnaire-based surveys only display a “snapshot” of subjective outcomes on a certain day and may not necessarily reflect a causal association. However, the observed improvements in QoL in our investigation are supported by previous research, documenting the beneficial effects of exercise and specifically HIIT on several self-reported outcomes [59,63]. Regarding physical activity and unsupervised exercise reporting, in particular, it must be considered that individuals, typically, rather tend to overestimate their physical activity levels [81]. We suppose, however, that the thorough explanation and briefing on how to record physical activity and home-based LOW-HIIT/exercise should have decreased the degree of potential errors.

It is a further limitation of this study that we did not include a passive control group. However, the benefits of physical exercise compared to an inactive lifestyle are already very well established based on decades of research. More specifically, a number of recent meta-analyses [82,83,84,85], including studies from our laboratory [17,18,19,20], have demonstrated that (LOW-)HIIT interventions elicit significantly greater improvements in VO_2max_ and cardiometabolic outcomes compared to no-training control conditions in both healthy and clinical populations. Thus, we felt that the inclusion of a passive control group without training would not have provided any essential new knowledge to the research field and that withholding of an established exercise modality would not have been ethically justifiable. We also note that we did not standardize participants’ diet throughout the intervention period. Thus, although there were neither significant differences in daily nutrition between the recording periods (T-1 vs. T-2, and T-2 vs. T-3) nor between the 3 sub-groups at T-3 (except for fat intakes in the HIIT- vs. EX-group) and no changes in body composition over time, it cannot be ruled out that potential within- or between-group variations in nutrition might have affected the results to some extent. However, we point out that it was the aim of our investigation to explore the effects of a worksite-based, LOW-HIIT program in a real-world setting without influencing our participants’ habitual daily nutrition. Moreover, a strict dietary standardization over a period of one year would not have been realizable under authentic conditions.

Finally, we acknowledge that the continuation of LOW-HIIT at home was strongly dependent on the availability of a cycle-ergometer, which constitutes a key bias in this study. Indeed, cycle ergometer-based exercise allows a very feasible implementation of our LOW-HIIT protocol because it is technically less demanding and probably better tolerated by previously untrained individuals compared to other exercise modalities, such as running or body weight exercises [48]. However, we note that LOW-HIIT is not exclusively limited to cycle ergometers, but can also be applied using other exercise modes. It has been reported, for example, that brief, vigorous stair climbing may be a readily accessible exercise modality to improve cardiorespiratory fitness in sedentary individuals [86]. Future studies may wish to further focus on investigating the potential health-related benefits that could arise from “equipment-free” LOW-HIIT protocols.

## 5. Conclusions

In summary, our data demonstrate that a 6-month, worksite-based, LOW-HIIT program resulted in improvements in VO_2max_, several cardiometabolic health outcomes and QoL in previously sedentary office workers. Study participants who continued unsupervised LOW-HIIT at home for 6 months during the COVID-19-related lockdown maintained VO_2max_ and QoL and further improved blood pressure values. Therefore, we conclude that LOW-HIIT appears to be an effective and extremely time-efficient exercise modality to mitigate the negative health impacts of COVID-19 pandemic-induced physical inactivity.

## Figures and Tables

**Table 1:** Mean values and standard deviations (SD) of participants’ (N = 71) cardiorespiratory fitness data pre- (T-1) and post-intervention (T-2).

Variable	T-1	T-2
Relative VO_2max_ (mL/kg/min)	34.2 ± 8.6	35.6 ± 7.5 **
Absolute VO_2max_ (L/min)	2.9 ± 0.8	3.0 ± 0.8 **
Relative peak power output (W/kg)	2.7 ± 0.7	3.0 ± 0.7 ***
Absolute peak power output (W)	223 ± 59	247 ± 61 ***
Power output at VT (W)	99 ± 41	141 ± 46 ***

VO_2max_, maximum oxygen uptake; VT, ventilatory threshold. **, and *** indicate *p* < 0.01, and *p* < 0.001 vs. T-1 in paired *t*-tests.

**Table 2:** Mean values and standard deviations (SD) of participants’ (N = 71) body composition and cardiometabolic data at baseline (T-1) and post-intervention (T-2).

Variable	T-1	T-2
Body weight (kg)	84.3 ± 18.5	83.8 ± 18.4
Body mass index (kg/m^2^)	26.9 ± 4.6	26.8 ± 4.8
Waist circumference, (cm)	89.3 ± 17.1	91.4 ± 13.4
Body fat mass (%)	30.0 ± 9.4	30.7 ± 8.9
Fat free mass (kg)	58.7 ± 12.2	58.0 ± 12.0
Resting heart rate (b/min)	89 ± 20	71 ± 10 ***
Systolic BP (mmHg)	131 ± 14	129 ± 13
Diastolic BP (mmHg)	90 ± 10	85 ± 8 ***
MAB (mmHg)	103 ± 11	99 ± 9 ***
Fasting glucose (mg/dL)	102 ± 25	101 ± 13
HbA_1c_ (%)	5.4 ± 0.6	5.2 ± 1.0 **
Triglycerides (mg/dL)	116 ± 54	109 ± 64
Total cholesterol (mg/dL)	222 ± 41	222 ± 38
HDL (mg/dL)	58 ± 14	59 ± 14
LDL (mg/dL)	145 ± 34	141 ± 30

BP, blood pressure; MAB, mean arterial blood pressure; HbA_1c_, glycosylated hemoglobin A_1c_; HDL, high-density lipoprotein cholesterol; LDL, low-density lipoprotein cholesterol. **, and *** indicate *p* < 0.01, and *p* < 0.001 vs. T-1 in paired *t*-tests and Wilcoxon test (only HbA_1c_), respectively.

**Table 3:** Mean values and standard deviations (SD) of participants’ (N = 71) self-reported outcomes at baseline (T-1) and post-intervention (T-2).

Variable	T-1	T-2
EQ-5D-5L (index)	0.96 ± 0.06	0.94 ± 0.09
EQ (VAS)	78 ± 12	83 ± 10 ***
Work Ability Index	41 ± 4	42 ± 4
PSQ-worries	21 ± 18	18 ± 14
PSQ-tension	30 ± 21	27 ± 17
PSQ-joy	67 ± 22	70 ± 22
PSQ-demands	39 ± 21	36 ± 18

EQ, EuroQol Group questionnaire; VAS, visual analogue scale; PSQ, Perceived Stress Questionnaire. *** indicates *p* < 0.001 vs. T-1 in Wilcoxon test.

**Table 4:** Mean values and standard deviations (SD) of participants’ (N = 57) age at follow-up (T-3), physical activity and nutritional intakes prior to the post-intervention (T-2) and follow-up examination (T-3).

Variable	HIIT-Group (N = 19)		EX-Group (N = 12)		NO-EX-Group (N = 26)	
	T-2	T-3	T-2	T-3	T-2	T-3
Age (yrs)	---	50 ± 9	---	52 ± 8	---	47 ± 12
Light PA (hours/week)	4.0 ± 2.0	2.9 ± 1.0 *	3.6 ± 1.6	3.0 ± 1.2	4.4 ± 2.1	3.4 ± 1.9 ***
Moderate PA (hours/week)	0.8 ± 0.8	0.3 ± 0.5 **	0.8 ± 0.7	0.2 ± 0.4 *	1.2 ± 0.8	0.4 ± 0.5 ***
MET hours/week	11.0 ± 5.5	6.1 ± 3.3 ***	10.9 ± 4.2	6.2 ± 3.1 **	13.6 ± 6.6	8.2 ± 4.3 ***
Energy intake(kcal)	2403 ± 858	2697 ± 1389	1946 ± 584	2051 ± 637	2174 ± 642	2062 ± 529
CHO intake (g)	246 ± 92	272 ± 137	209 ± 71	210 ± 75	219 ± 79	210 ± 57
Fat intake (g)	101 ± 43	110 ± 63	70 ± 29 +	79 ± 31 +	91 ± 28	83 ± 30
Protein intake (g)	96 ± 41	114 ± 62	77 ± 30	89 ± 38	87 ± 32	89 ± 29

PA, physical activity; MET, metabolic equivalent; CHO, carbohydrates. *, **, and *** indicate *p* < 0.05, *p* < 0.01, and *p* < 0.001 vs. T-2, and + indicates *p* < 0.05 vs. HIIT-group, respectively, in 2-way repeated measures ANOVAs.

**Table 5:** Mean values and standard deviations (SD) of participants’ (N = 57) cardiorespiratory fitness data at post-intervention (T-2) and follow-up (T-3).

Variable	HIIT-Group (N = 19)		EX-Group (N = 12)		NO-EX-Group (N = 26)	
	T-2	T-3	T-2	T-3	T-2	T-3
Relative VO_2max_ (mL/kg/min)	35.0 ± 5.7	35.0 ± 6.1	36.7 ± 6.5	32.9 ± 7.6 ***	37.5 ± 8.2	31.6 ± 8.2 ***
Absolute VO_2max_ (L/min)	3.0 ± 0.6	2.9 ± 0.6	2.7 ± 0.6	2.4 ± 0.7 ***	3.1 ± 0.6	2.5 ± 0.9 ***
Relative peak power output (W/kg)	2.9 ± 0.6	2.9 ± 0.5	3.2 ± 0.6	3.1 ± 0.6	3.0 ± 0.7	2.9 ± 0.7
Absolute peak power output (W)	253 ± 46	241 ± 40	243 ± 60	238 ± 62	251 ± 65	241 ± 60
Power output at VT (W)	137 ± 47	122 ± 30	154 ± 45	112 ± 47 ***	146 ± 47	110 ± 37 ***

VO_2max_, maximum oxygen uptake; VT, ventilatory threshold. *** indicates *p* < 0.001 vs. T-2 in 2-way repeated measures ANOVAs.

**Table 6:** Mean values and standard deviations (SD) of participants’ (N = 57) body composition and cardiometabolic data at post-intervention (T-2) and follow-up (T-3).

Variables	HIIT-Group (N = 19)		EX-Group (N = 12)		NO-EX-Group (N = 26)	
	T-2	T-3	T-2	T-3	T-2	T-3
Body weight (kg)	87.5 ± 18.8	86.3 ± 18.4	73.4 ± 13.3	75.4 ± 14.0	83.7 ± 20.2	83.4 ± 19.2
Body mass index (kg/m^2^)	27.8 ± 4.7	27.5 ± 5.1	24.9 ± 3.1	25.0 ± 3.2	25.9 ± 4.8	25.8 ± 4.6
Waist circumference, (cm)	96.8 ± 14.1	95.3 ± 12.9	87.2 ± 11.5	86.8 ± 10.1	89.8 ± 13.7	89.7 ± 13.5
Body fat mass (%)	29.9 ± 7.7	29.1 ± 7.9	27.9 ± 6.4	28.0 ± 6.6	29.6 ± 9.9	29.5 ± 9.1
Fat free mass (kg)	60.5 ± 10.8	60.4 ± 10.7	54.4 ± 11.4	54.4 ± 11.0	58.6 ± 13.2	58.3 ± 13.1
Resting heart rate (b/min)	73 ± 12	71 ± 9	69 ± 8	70 ± 12	72 ± 9	83 ± 10 ***
Systolic BP (mmHg)	135 ± 12	126 ± 12 **	126 ± 15	123 ± 13	126 ± 12	127 ± 11
Diastolic BP (mmHg)	89 ± 8	84 ± 8 *	85 ± 9	83 ± 7	84 ± 6	86 ± 7
MAB (mmHg)	102 ± 9	98 ± 8 *	98 ± 11	97 ± 9	98 ± 7	101 ± 8 *
Fasting glucose (mg/dL)	100 ± 10	101 ± 11	102 ± 19	108 ± 21	101 ± 13	105 ± 19
HbA_1c_ (%)	5.4 ± 0.3	5.4 ± 0.3	5.5 ± 0.5	5.5 ± 0.4	5.3 ± 0.4	5.4 ± 0.5
Triglycerides (mg/dL)	120 ± 68	120 ± 91	85 ± 32	94 ± 44	108 ± 68	119 ± 71
Total cholesterol (mg/dL)	226 ± 36	228 ± 39	215 ± 28	221 ± 37	222 ± 45	228 ± 45
HDL (mg/dL)	54 ± 11	57 ± 11	62 ± 15	62 ± 17	62 ± 18	62 ± 16
LDL (mg/dL)	147 ± 29	149 ± 33	134 ± 30	140 ± 34	139 ± 33	146 ± 34

BP, blood pressure; MAB, mean arterial blood pressure; HbA_1c_, glycosylated hemoglobin A_1c_; HDL, high-density lipoprotein cholesterol; LDL, low-density lipoprotein cholesterol. *, **, and *** indicate *p* < 0.05, *p* < 0.01, and *p* < 0.001 vs. T-2 in 2-way repeated measures ANOVAs.

**Table 7:** Mean values and standard deviations (SD) of participants’ (N = 57) self-reported outcomes at post-intervention (T-2) and follow-up (T-3).

Variable	HIIT-Group (N = 19)		EX-Group (N = 12)		NO-EX-Group (N = 26)	
	T-2	T-3	T-2	T-3	T-2	T-3
EQ-5D-5L (index)	0.92 ± 0.07	0.93 ± 0.07	0.96 ± 0.06	0.94 ± 0.08	0.95 ± 0.04	0.94 ± 0.06
EQ (VAS)	81 ± 10	80 ± 13	86 ± 8	85 ± 8	85 ± 7	80 ± 11 *
Work Ability Index	42 ± 4	42 ± 3	43 ± 4	43 ± 3	42 ± 4	43 ± 3
PSQ-worries	20 ± 15	21 ± 16	23 ± 16	19 ± 16	14 ± 18	16 ± 13
PSQ-tension	32 ± 22	35 ± 22	27 ± 11	34 ± 22	26 ± 12	30 ± 22
PSQ-joy	64 ± 28	60 ± 27	75 ± 17	71 ± 23	73 ± 20	70 ± 17
PSQ-demands	45 ± 19	43 ± 20	37 ± 16	42 ± 23	36 ± 18	34 ± 22
PSQ-total	34 ± 19	35 ± 18	27 ± 13	32 ± 21	27 ± 11	27 ± 15

EQ, EuroQol Group Questionnaire; VAS, visual analogue scale; PSQ, Perceived Stress Questionnaire. * indicates *p* < 0.05, vs. T-2 in Friedman two-way analyses by ranks.

## Data Availability

The datasets generated and analyzed during the current study are not publicly available but are available from the corresponding author on reasonable request.
